# Heart failure and chronic obstructive pulmonary disease. A combination not to be underestimated

**DOI:** 10.1007/s10741-025-10566-3

**Published:** 2025-10-07

**Authors:** Damiano Magrì, Emiliano Fiori, Piergiuseppe Agostoni, Michele Correale, Massimo Piepoli, Savina Nodari, Matteo Beltrami, Stefania Paolillo, Pasquale Perrone Filardi, Alberto Palazzuoli

**Affiliations:** 1 Department of Clinical and Molecular Medicine, Sapienza University of Rome, Rome, Italy; 2 Centro Cardiologico Monzino, IRCCS, Milan, Italy; 3 Cardiovascular Section, Department of Clinical Sciences and Community Health, University of Milano, Milan, Italy; 4 Cardiothoracic Department, Ospedali Riuniti University Hospital, Foggia, Italy; 5 Clinical Cardiology, IRCCS Policlinico San Donato, San Donato Milanese, Milan, Italy; 6 Department of Medical and Surgical Specialties, Radiological Sciences and Public Health, University of Brescia Medical School, Brescia, Italy; 7 Arrhythmia and Electrophysiology Unit, Careggi University Hospital, Florence, Italy; 8 Department of Advanced Biomedical Sciences, Cardiology Unit, University Federico II Naples, Naples, Italy; 9 Cardiovascular Diseases Unit, Cardio-Thoracic and Vascular Department, S. Maria Alle Scotte Hospital, University of Siena, Siena, Italy

**Keywords:** Heart failure, Lung disease, Cardiopulmonary exercise test, Cardiopulmonary interaction

## Abstract

Chronic obstructive pulmonary disease (COPD) and heart failure (HF) frequently coexist and interact through complex and bidirectional hemodynamic mechanisms that amplify symptoms’ burden and complicate clinical management. The present review explores the impact of COPD across the HF spectrum, particularly in HF with preserved ejection fraction (HFpEF), where comorbidities, such as COPD, exert a dominant role in disease expression. COPD-induced hyperinflation reduces cardiac preload and increases right ventricular afterload, while HF-related congestion impairs pulmonary function and gas exchange, illustrating a tight cardiorespiratory coupling. Diagnostic challenges stem from overlapping symptoms and the limited specificity of biomarkers, such as natriuretic peptides, especially in HFpEF. Cardiopulmonary exercise testing (CPET) emerges as a valuable tool for distinguishing between cardiac and pulmonary limitations and guiding individualized treatment strategies. From a therapeutic standpoint, *β*1-selective blockers are not only safe in COPD patients but are pivotal in those with HF with reduced ejection fraction (HFrEF), where they have been demonstrated to improve survival and reduce both HF and COPD exacerbations. Concerns regarding bronchodilator safety in HF remain largely theoretical, with current evidence supporting their continued use when clinically indicated. Ultimately, optimal care for patients with coexisting COPD and HF requires a phenotype-specific approach, incorporating insights from pathophysiology, diagnostic innovation, and evidence-based pharmacotherapy to improve outcomes in this challenging patient population.

**Supplementary Information:**

The online version contains supplementary material available at 10.1007/s10741-025-10566-3.

## Introduction

Chronic obstructive pulmonary disease (COPD) and heart failure (HF) are two global pandemics [1, 2], with a prevalence of approximately 1–3% and 10%, respectively. Their prevalence is expected to rise in the coming years, and both of them are associated with severe morbidity, impaired quality of life, and high mortality rates, placing a substantial financial and organizational burden on healthcare systems worldwide.

Their coexistence presents significant clinical challenges, particularly across the diverse spectrum of HF ejection fractions. The evolving understanding of HF has revealed a broader range of phenotypes, i.e., HF with mildly reduced (HFmrEF) and the one with preserved ejection fraction (HFpEF) [3]. Despite its increasing diagnosis, there is still a lack of comprehensive therapeutic guidance in current clinical guidelines, especially complicating the management of those HF patients where disease development and clinical manifestations are more heavily influenced by comorbidities [4]. In such a context, COPD has one of the most profound impacts, exacerbating symptoms, accelerating disease progression, and complicating therapeutic decisions. COPD contributes to systemic inflammation, pulmonary hypertension, and impaired filling pressures, all of which interact with HF’s pathophysiology [5] to worsen functional capacity and quality of life. This underscores the need for a deeper understanding of how COPD distinctly affects each HF phenotype to guide more targeted and effective treatment strategies.

This review explores the multifaceted impact of COPD across the HF spectrum, focusing on four critical domains: (1) the epidemiology and prognosis of COPD and HF comorbidity across EF categories, (2) the pathophysiological interactions that intensify symptoms and functional limitations, (3) the diagnostic challenges posed by overlapping clinical features, and (4) the persistent therapeutic gaps, aiming to separate longstanding myths from evidence-based practices.

## Epidemiology of COPD along the HF spectrum

### Impact of HF in COPD patients

**Fig. 1:**
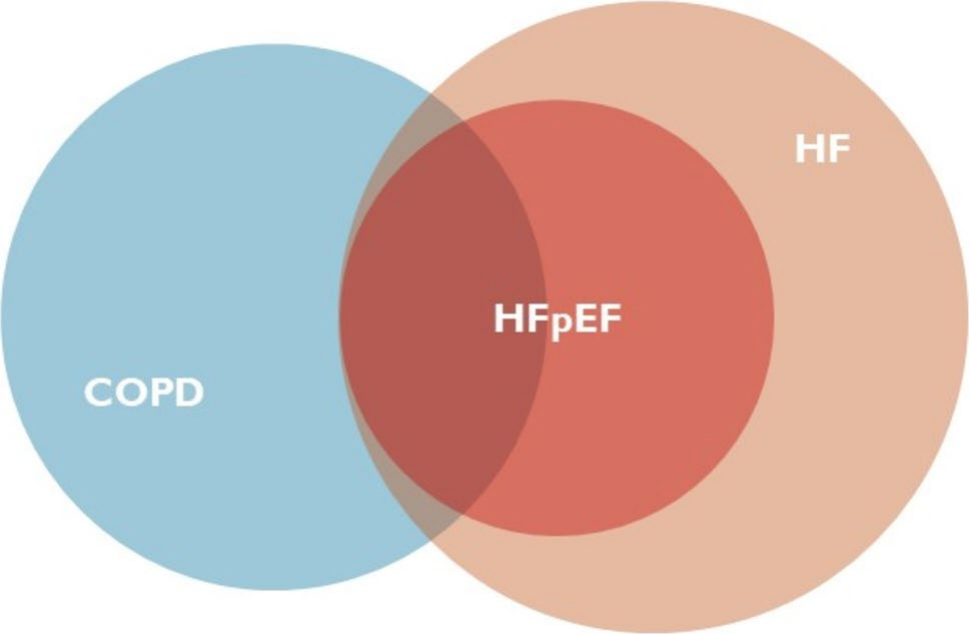
Prevalence and clinical overlap of HF among COPD patients. Approximately one in five patients with COPD also has HF. Among those with both conditions, about 70% present with the preserved ejection fraction phenotype (HFpEF), highlighting the significant clinical overlap and the predominance of HFpEF in this population: COPD, chronic obstructive pulmonary disease

Among patients with COPD, the incidence of diagnosed HF is around 1.2 per 100 person-years, stable in the last 20 years [6]. Prevalence of HF among COPD patients ranges from 11.1 to 21.1% [7, 8], and 70% of patients with COPD and HF present the preserved EF phenotype [9] (Fig. 1). There is substantial evidence that HF comorbidity increases COPD-related rehospitalization and all-cause mortality among COPD patients. The effect of HF comorbidity may differ depending on COPD phenotype, HF type, or HF severity [10]. A cohort study based on longitudinal American registries explored the differential impact of HF EF phenotype in the COPD population [9]. All-cause hospitalization did not differ across EF groups; however, patients with COPD and HFrEF had a greater risk of HF-specific hospitalization and mortality than patients with COPD and HFpEF. Conversely, patients with COPD and HFrEF had a lower risk of acute exacerbation of COPD (AECOPD) than those with COPD and HFpEF. Outcomes in patients with comorbid COPD and HFpEF were largely driven by COPD. This evidence is confirmed by the fact that the burden of comorbidities contributes to symptoms more in HFpEF than in HFrEF [11], and among them, COPD is associated with more prominent reductions in quality of life. Interestingly, over a 10-year study period, no difference in cardiovascular mortality in patients with COPD and comorbid HF has been described, in contrast with the trend seen in the wider COPD population toward decreasing cardiovascular deaths [6]. This underlines that managing comorbid COPD and HF remains a clear unmet need in contemporary chronic disease care, as the superimposition of HF on COPD significantly influences the clinical and prognostic course—HFpEF is associated with a worsening respiratory trajectory, whereas HFrEF is linked to more HF and cardiovascular (CV) events.

### Impact of COPD in HF patients

Data describing the relationship between COPD and HF from the cardiovascular research perspective can be distinguished, whether they are derived from registries or clinical trials. In the most recent study conducted on a European registry, the prevalence of COPD in HF patients was 13%. Patients with versus without COPD were more likely to have HFpEF versus HF with mid-range EF (HFmrEF) and HFrEF (16%, 12%, and 11%, respectively) [12]. Patients with HF and concomitant COPD were older, more likely to be female, had a higher burden of cardiovascular (CV) and non-CV comorbidities, and more severe HF. COPD was associated with higher risks of CV death, first and recurrent HF hospitalization (HFH), all-cause death, and non-CVD across the EF spectrum. A statistically significant interaction between EF phenotypes and COPD for the outcomes of first and repeated HF hospitalizations was found, with the highest risk in HFmrEF and HFpEF, which is consistent with a higher prognostic role of CV but also non-CV comorbidities with higher EF. A recent focused analysis on HFmrEF confirmed a similar COPD prevalence (12%) and the independent additive risk of major CV and non-CV outcomes [13]. These results reinforce and expand the established knowledge on the deleterious prognostic effect of COPD in the HF population [14]. Table 1 summarizes the impact of COPD across EF categories of HF in the most recent randomized clinical trials (RCT) [15–21]. The prevalence of COPD in these contemporary but selected populations ranges from 11.1 to 14.2%. Patients with COPD and HF, independently of EF, present a poorer quality of life and worse outcomes. Interestingly, these post hoc analyses consistently demonstrate a preserved benefit of GDMT irrespective of the presence of COPD. A meta-analysis of three post hoc RCTs and eight observational studies underscores the impact of COPD on hospitalization and mortality in patients with HFpEF [22]. Given the paucity of effective therapies for HFpEF, more precise differentiation between cardiac and respiratory symptoms may provide an opportunity to mitigate the risk of AECOPD. The elevated risk of mortality and HF hospitalization in patients with concomitant COPD and HFrEF further underscores the imperative to optimize guideline-directed medical therapy for HFrEF in this population.

**Table 1:** Prevalence and clinical and prognostic impacts of COPD among HF patients: insights from randomized controlled trials

Reference (n)	*Trial*	*Patients (N)*	*Medication*	*COPD prevalence*	*NYHA III-IV prevalence*	*NT-proBNP (pg/ml)*	*KCCQ*	*Primary outcome*	*Event rate (per 100 pts/y) and HR*	Treatment interaction
***HFrEF***										
Su E. Yeoh et al. (2022) [11]	**RALES EMPHASIS-HF** (pooled)	4397	Spironolactone/Eplerenone	14.2%	37.4 vs 34.2 (*p* = 0.19)	N.A	N.A	Composite of HF hospitalization or CV death	25.2 vs 19.9 HR = 1.25 (1.08–1.44) *p* < 0.001	Consistent benefit (*p* = 0.93)
S. Ehteshami-Afshar et al. (2021) [12]	**PARADIGM-HF**	8399	Sacubitril/valsartan	12.9%	37 vs 23 (*p* < 0.001)	1741 (949–3503) vs 1591 (882–3171), *p* = 0.011	73.4 (56.1–87.5) vs 81.2 (64.6–92.7), *p* < 0.001	Composite of CV death or first HF hospitalization	15.16 vs 11.33 HR = 1.33 (1.18–1.50) *p* < 0.001	Consistent benefit (*p* = 0.171)
P. Dewan et al. (2020) [13]	**DAPA-HF**	4744	Dapagliflozin	12.3%	42.6 vs 31.1 (*p* < 0.001)	1574 (893–2807) vs 1418 (850–2616) *p* = 0.021	71 (53–85) vs 79 (60–93), *p* < 0.001	Composite of worsening HF or CV death	18.9 vs 13.0 HR = 1.44 (1.21–1.72) *p* < 0.001	Consistent benefit (*p* = 0.47)
***HFpEF***										
S. H. R. Ramalho et al. (2020) [14]	**TOPCAT**	3445	Spironolactone	11.8%	43.0 vs 33.0 (*p* < 0.001)	993 (57–1804) vs 955 (580–2054) *p* = 0.89	N.A	Composite of HF hospitalization or CV death	15.0 vs 10.5 HR = 1.37 (1.13–1.66) *p* = 0.001	Reduction in CV (*p* = 0.01) and all-cause mortality (*p* = 0.02) among COPD patients
L. Mooney et al. (2021) [15]	**PARAGON-HF**	4791	Sacubitril/valsartan	14%	24.0 vs 19.0 (*p* = 0.003)	913 (453–1606) vs 887 (467–1647), *p* = 0.67	69.3 (55.0–82.8) vs 76.0 (60.9–88.5), *p* < 0.001	Composite of total HF hospitalizations and CV death	24.30 vs 12.07 HR = 1.78 (1.48–2.13) *p* < 0.001	Consistent absence of benefit (*p* = 0.66)
J. H. Butt et al. (2023) [16]	**DELIVER**	6261	Dapagliflozin	11.1%	34.5 vs 23.5 (*p* < 0.001)	1433 (987–2212) vs 1398 (957–2210) *p* = 0.68	65.3 ± 21.5vs 70.6 ± 22.2 *p* < 0.001	Composite of worsening HF or CV death	13.3 vs 8.1 HR = 1.63 (1.39–1.91) *p* < 0.001	Consistent benefit (*p* = 0.98)
***HFrEF/pEF***										
S. Von Haehling et al. (2024) [17]	**EMPEROR-reduced** **EMPEROR-preserved** (pooled)	9718	Empagliflozin	12.7%	19.4 vs 28.4 (*p* < 0.001)	N.A	N.A	Composite of first HF hospitalization or CV death	HR = 1.52, (1.28–1.80) *p* < 0.0001	Consistent benefit (*p* = 0.50)

The contemporary presence of COPD in HF patients is associated with a worsened functional status, as reflected by a higher prevalence of NYHA Classes III–IV and lower KCCQ scores. This impact appears more pronounced in HFpEF compared to HFrEF populations. NT-proBNP levels do not appear to be significantly influenced by COPD in HFpEF populations. In contrast, in HFrEF studies, patients with COPD showed significantly elevated NT-proBNP levels. Across HF phenotypes, COPD presence is consistently associated with worse prognosis, reflected in higher event rates and hazard ratios for primary outcomes such as HF hospitalization and cardiovascular death. Importantly, the benefit of GDMT remains consistent regardless of COPD status

*CV* cardiovascular, *COPD* chronic obstructive pulmonary disease, *HFrEF* heart failure with reduced ejection fraction, *HFpEF* heart failure with preserved ejection fraction, *NYHA* New York Heart Association functional class, *NT-proBNPN*-terminal pro brain-derived natriuretic peptide, *KCCQ* Kansas City cardiomyopathy questionnaire

## Pathophysiological interactions between COPD and heart failure

COPD and HF share complex and bidirectional pathophysiological interactions, with each condition exacerbating the severity of the other. This interplay contributes to increased symptom burden and exercise limitation in comorbid patients.

### How HF aggravates COPD

One of the primary mechanisms of resting and/or exertional dyspnea in HF is pulmonary congestion, which may be associated with varying degrees of airway disorder. Volume overload in HF leads to a reduction in lung volumes and airway caliber [23]. In HFpEF, left atrial pressure (LAP) may be slightly elevated at rest but can double during mild exercise or when in the supine position due to fluid shifts. This elevation in filling pressures contributes to a reduction in airway cross-sectional area and an increase in bronchial reactivity due to bronchial mucosa vascular engorgement [24]. Volume shifts and interstitial edema are indeed strong predictors of airway obstruction in COPD patients [25].

The alveolar-capillary unit in HF undergoes unfavorable remodeling due to impaired alveolar clearance [26] and chronic or acute hemodynamic stress [27]. During exercise in HF patients, there is a volume shift from the vascular to the interstitial space, confirmed by an increase in B-lines (comets) on lung ultrasound, without significant changes in diffusion. However, during recovery, interstitial fluid moves toward the alveolar-capillary membrane, clearing the interstitial space but worsening gas diffusion [28]. These changes lead to a ventilation/perfusion (V/Q) mismatch and hypoxemia. Hypoxemia, in turn, induces pulmonary vasoconstriction, further aggravating dead space ventilation in COPD patients [29].

In patients with HF, exertional dyspnea is commonly attributed to basal ventilatory restriction, which is particularly pronounced in dilated cardiomyopathy and HFrEF [30]. This is further aggravated by increased breathing effort resulting from an exaggerated ventilatory response. Hyperventilation in HF is driven not only by ventilation–perfusion mismatch but also by sympathetic activation of chemoreceptors and by metaboreflex stimulation from skeletal and respiratory muscles [31]. Both HFrEF and HFpEF are characterized by muscle wasting and cachexia [32], further exacerbating the respiratory muscle fatigue already present in COPD and significantly contributing to symptomatic burden [33].

### How COPD aggravates HF

COPD is characterized by variable degrees of airway flow obstruction and lung parenchymal rarefaction (emphysema). Among COPD patients without an established diagnosis of cardiovascular disease, more than half present with grade II diastolic dysfunction, suggesting a high prevalence of elevated left ventricular (LV) filling pressure and possibly HFpEF [34]. However, abnormal filling patterns observed in echocardiography may not necessarily indicate high LV preload but rather reflect reduced filling pressure patterns.

A landmark study on 2,816 patients with severe COPD demonstrated a linear relationship between lung emphysema percentage and reductions in left ventricular end-diastolic volume, stroke volume (SV), and cardiac output (CO) [35]. Greater emphysema severity was also associated with a reduced total pulmonary vein area [36] and smaller right ventricular (RV) volumes and SV [37], supporting a mechanism of upstream circulatory impairment leading to ventricular underfilling.

Airflow obstruction due to reduced parenchymal recoil leads to progressive air trapping. During exercise, this condition is further aggravated by dynamic hyperinflation, which generates positive intrathoracic pressures and reduces venous return [38, 39]. Chronic air trapping and dynamic hyperinflation ultimately alter diaphragm configuration, flattening its dome-shaped structure. The diaphragm plays a critical role in modulating venous return and biventricular afterloads during respiratory cycles. Severe diaphragmatic atrophy, commonly observed in both HF and COPD, further contributes to impaired biventricular filling [40].

Reduced RV preload is accompanied by an abnormal increase in afterload, both at rest and particularly during exercise. The prevalence of precapillary pulmonary hypertension (PH) rises with COPD severity [41]. Even in the absence of resting PH, pulmonary pressures rise steeply during exercise because of impaired cardiac output adaptation (mPAP/CO slope > 3) [42], a condition linked to reduced functional capacity [43]. Alveolar disruption and pulmonary vascular remodeling with pulmonary arterial thickening in emphysema result in stable increases in pulmonary vascular resistance (PVR). During exercise, this condition is exacerbated by the pulsatile load of the LAP V-wave, which augments pulmonary arterial elastance and RV afterload.

**Fig. 2:**
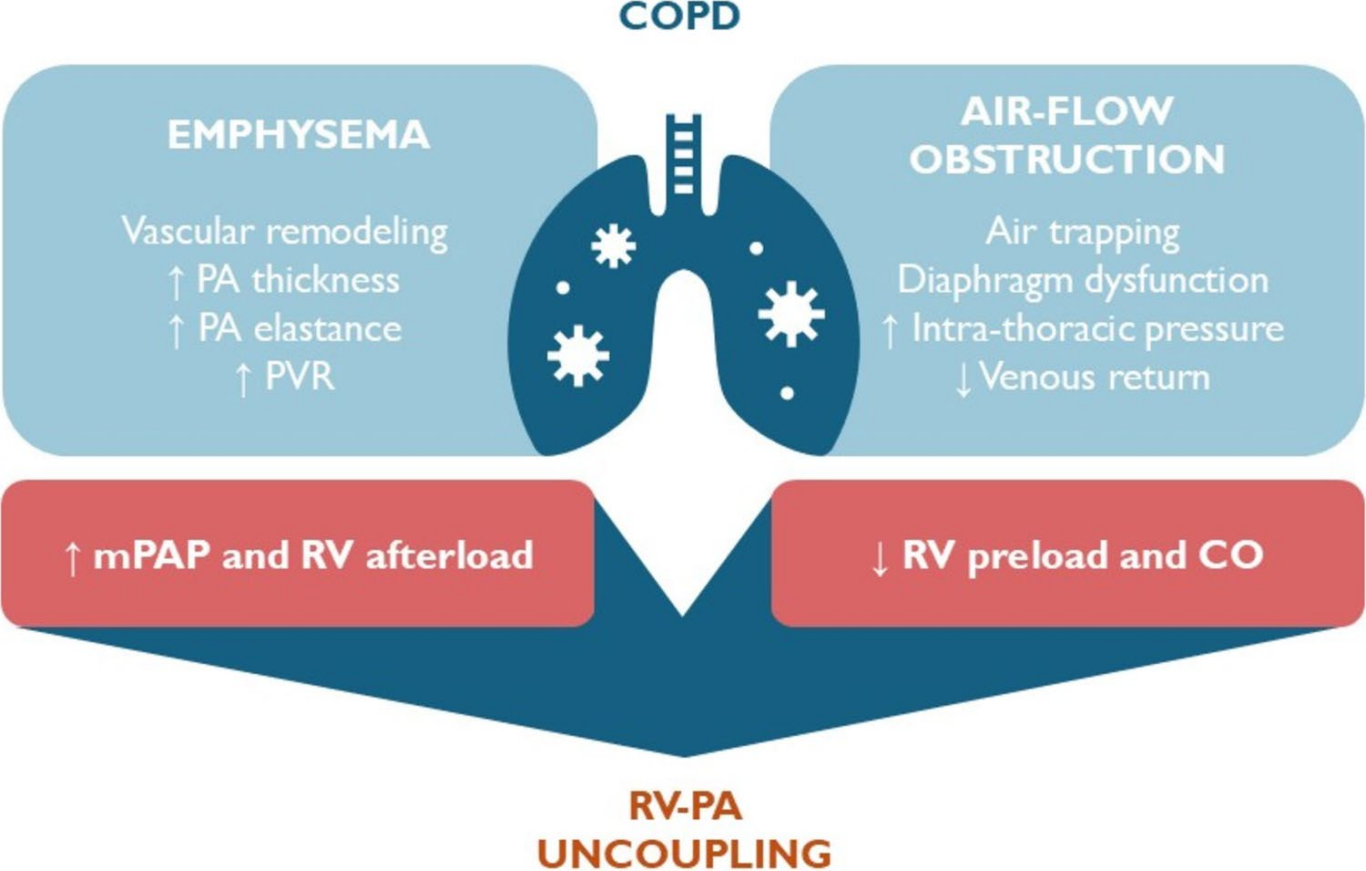
Emphysema and air-flow obstruction interplay in the pathogenesis of cardiac impairment among COPD patients. Lung parenchymal rarefaction in COPD is associated with pulmonary vascular remodeling and increased pulmonary vascular resistance (PVR). Airflow obstruction, whether present at rest or during exertion, together with chronic diaphragmatic dysfunction, contributes to reduced venous return. The combined effect of increased lung vascular barrage and diminished right ventricular (RV) preload may lead to RV to pulmonary artery (PA) uncoupling and blunt left ventricular (LV) output reserve during exercise; COPD, chronic obstructive pulmonary disease; PA, pulmonary artery; PVR, pulmonary vascular resistance; mPAP, mean pulmonary artery pressure; CO, cardiac output

A unifying hypothesis suggests that COPD patients experience altered central hemodynamics during exercise [44], characterized by reduced biventricular preload and increased RV afterload, potentially leading to RV-pulmonary artery uncoupling and reduced CO[45]. In the presence of established diastolic dysfunction, such as in HFpEF, the reduction in volume return from the right-sided circulation, coupled with typical chronotropic incompetence [46], may impair the heart’s ability to increase CO sufficiently to match peripheral metabolic demands during exercise (preload failure) (Fig. 2).

## Differential diagnosis

COPD can produce or obscure nearly every symptom and sign associated with HF. Exertional breathlessness, nocturnal cough, and paroxysmal nocturnal dyspnea are common to both conditions, and no qualitative features of dyspnea are unique to HF. COPD can mimic HF or exacerbate underlying cardiovascular dysfunction, which is particularly concerning in HFpEF [47]. The echocardiographic alterations in HFpEF are more subtle compared to HFrEF, and transthoracic echocardiography may be impeded by poor acoustic windows due to the pathological changes associated with COPD in up to 50% of patients [48].

### Natriuretic peptides (NPs)

Both B-type natriuretic peptide (BNP) and N-terminal pro-BNP (NT-proBNP) are valuable for ruling out HF in acute dyspnea and confirming chronic HF [49]. However, their diagnostic accuracy in patients with concurrent COPD is less established.

NP levels are elevated in patients with stable COPD who do not exhibit signs of left-sided HF, which negatively impacts prognosis [50]. Identifying HFpEF is already controversial and problematic, and the presence of COPD further complicates this process. A significant subset of HFpEF patients exhibit circulatory congestion exclusively during exercise while maintaining normal NP levels at rest [51], thereby reducing the rule-in accuracy of NP testing [52]. The overlap of COPD and HFpEF diminishes the overall diagnostic utility of NPs, as pulmonary diseases significantly affect both the positive and negative predictive values of the test [53]. Patients with HFpEF tend to be older and have a higher burden of non-respiratory comorbidities, both of which independently influence NP levels and should be considered when interpreting test results and threshold values [54]. By contrast, patients with HFrEF typically exhibit higher NP levels, even in the presence of COPD [53]. Unlike HFpEF, NP levels maintain a reliable negative predictive value for ruling out left ventricular systolic dysfunction in both stable COPD and during COPD exacerbations [55].

**Fig. 3:**
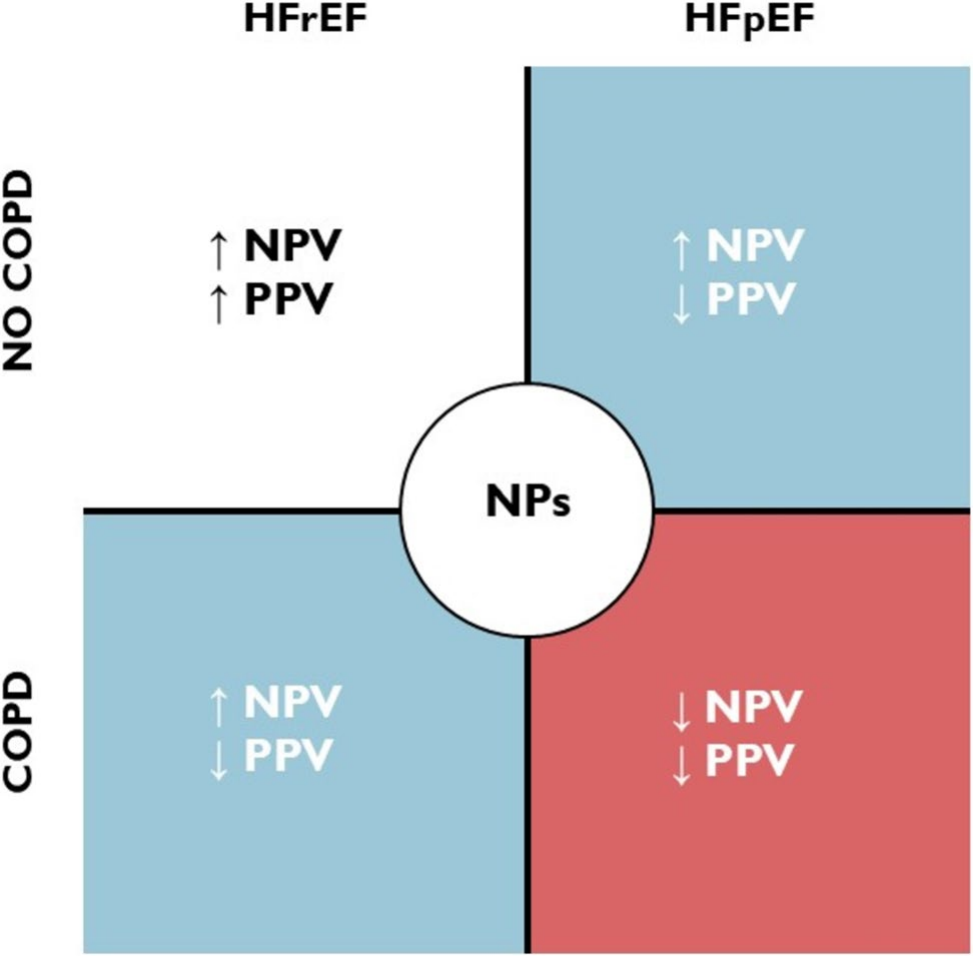
Impact of COPD on NPs' diagnostic accuracy in HF; NPV, negative predictive value; PPV, positive predictive value

Overall, COPD represents an important confounding factor in the interpretation of natriuretic peptide (NP) testing, particularly in the diagnostic workup of HFpEF (Fig. 3). Additional research is needed to establish and validate specific cut-off values that could optimize the diagnostic/prognostic accuracy of NPs in patients with coexisting COPD and HF.

### Pulmonary function tests (PFTs)

The differential diagnosis of COPD in HF patients is often based on clinical judgment, considering risk factors such as smoking habit, medication history, and physical examination. However, cardiologists frequently attribute symptom progression to a known disease rather than a potentially undiagnosed condition with a similar presentation [56]. Although spirometry is recommended for diagnosing COPD, it is only performed in approximately 30% of patients with HF and suspected (or presumed) COPD [57]. Due to this underutilization, about 30% of chronic HF patients are misdiagnosed with COPD despite having no confirmed airflow obstruction, while another 30% have undetected airflow limitation [58].

The diagnostic utility of spirometry in HF is affected by both acute and stable conditions. Acute HF is characterized by volume overload, which can lead to bronchial compression, vascular engorgement, and hyperreactivity, thereby narrowing the airways. A landmark study demonstrated that airway obstruction in HF is a dynamic phenomenon [59], with complete resolution in half of the patients when spirometry was repeated under stable conditions. A recent study conducted on stable HF patients described an inverse linear correlation between FEV1 reduction and NT-proBNP and pulmonary pressure elevation, thus reinforcing the framework of a volume-dependent obstructive component in HF [60]. In chronic HF, by contrast, pulmonary function is primarily characterized by a restrictive pattern due to cardiomegaly [60] and fibrotic changes in the pulmonary interstitium. The proportional reduction in FEV1 and FVC (10–20%) [61] can pseudo-normalize the obstructive defect, leading to underdiagnosis of COPD or underestimation of its severity. Although no specific recommendations exist for spirometry in HF, the test should ideally be performed under euvolemic and stable conditions to avoid overdiagnosis [57]. There is no significant difference in the applicability, interpretation, or performance of spirometry between HFrEF and HFpEF, as lung congestion remains the common pathway leading to functional airway obstruction in both conditions.

Hyperinflation and air trapping compromise proper exhalation and are hallmarks of COPD. Unlike spirometry, body plethysmography allows for the quantification of absolute total lung capacity (TLC) and residual volume (RV), making it a superior method for verifying air trapping. Since pulmonary congestion does not influence air trapping [59], lung volume measurements can improve diagnostic accuracy in differentiating COPD from HF. Consequently, body plethysmography with RV measurement should be considered a second-line test for diagnosing COPD in HF patients [47] when spirometry is inconclusive or a dynamic component related to subclinical congestion is suspected.

### Cardiopulmonary exercise testing (CPET)

Comprehensive cardiopulmonary functional assessment is provided by CPET, which integrates exercise testing with the analysis of respiratory gas volumes and composition. In HF patients, minute ventilation (VE) increases disproportionately relative to carbon dioxide production (VCO_2_) due to ventilation/perfusion (V/Q) mismatch, increased dead space fraction (dead space/tidal volume), and reduced arterial CO_2_ tension [31].

Recent data suggest that the VE/VCO_2_ slope does not significantly differ among patients with HF, COPD, or coexisting HF and COPD. However, a higher dead space (VD) load may explain the elevated intercept of the VE/VCO_2_ slope observed when COPD is present [62], regardless of HF status. Since increased VD is a primary pathophysiological abnormality in COPD but only a secondary feature in HF, a positive VE intercept (VEint ≥ 2.6–4.07 L/min) has emerged as a promising tool for identifying COPD as an HF comorbidity [63] across the whole spectrum of EF [64].

Apart from its established value in prognostication, CPET should be considered as an additional step in the differential diagnostic pathway of a stable patient with dyspnea and HF, to confirm or exclude the coexistence of COPD.

## Treatment interactions

In patients with coexisting COPD and HF, achieving bronchodilation through *β*2-receptor stimulation while simultaneously reducing overall sympathetic activity via *β*1-receptor blockade is both beneficial but appears to be opposing therapeutic goals.

### β-blockers

**Fig. 4:**
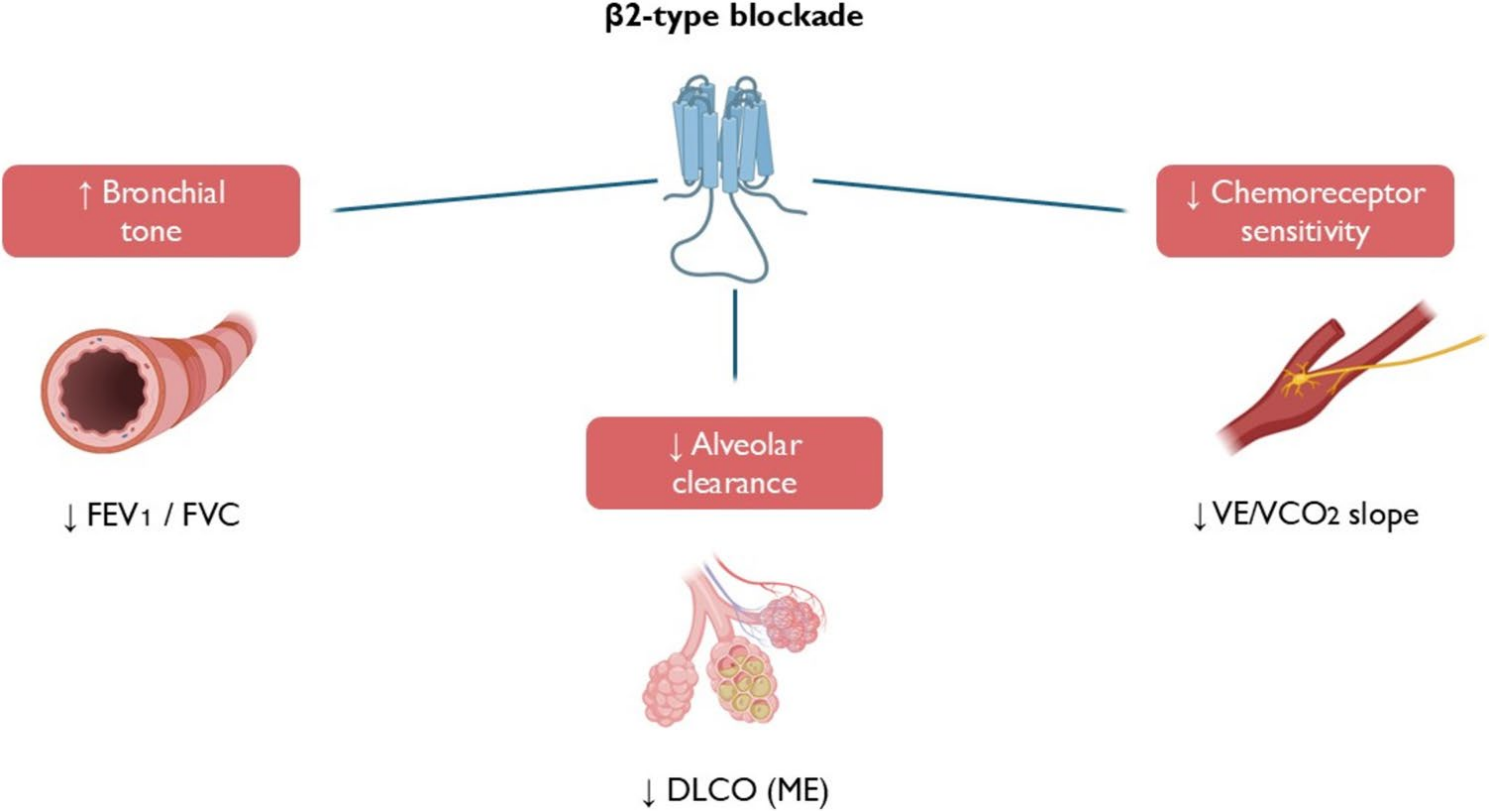
Multifaceted impact of non-selective *β*-blockers on respiratory function. Compared to *β*1-selective agents (e.g., bisoprolol), non-selective *β*1/*β*2-blockers (e.g., carvedilol) are associated with increased bronchial tone, reduced alveolar clearance, and attenuated chemoreceptor sensitivity, which may improve ventilatory efficiency during exercise. FEV1, forced expiratory volume in 1 s; FVC, functional vital capacity; DLCO, diffusing capacity of the lungs for carbon monoxide; ME, interstitial alveolar membrane; VE/VCO2, ventilatory efficiency slope

The main concern regarding the use of *β*-blockers in COPD patients derives from the possible class effect on the bronchial smooth muscle *β*2-type receptors, with the induction of bronchoconstriction and worsening air-flow limitation. However, nearly 90% of *β*2-type receptors of the respiratory system are located on the alveolar cells, where they regulate alveolar fluid clearance, with consequent effects on lung diffusing capacity. In addition, *β*-receptors are present at the level of chemoreceptors and ergoreceptors, where they modulate their sensitivity and can influence the ventilatory response to exercise [65]. Non-selective *β*-blockers can impact respiratory function at different levels (Fig. 4). Carvedilol is a non-selective *β*1–*β*2-type receptor blocker indicated in the treatment of HFrEF. Compared to bisoprolol (*β*1-selective), carvedilol reduces lung diffusion capacity (DLCO) due to changes in active membrane transport, which is under alveolar *β*2-receptor control [66]. The effect of *β*2-receptor blockade on airway function has been tested in several trials comparing carvedilol and *β*1-selective blockers in comorbid HF and COPD patients [67–69]. Carvedilol is associated with a significant reduction in FEV1 and FEV1/FVC ratio compared to bisoprolol, nebivolol, or metoprolol, which present a neutral effect. Carvedilol reduces hyperventilation and allows for better ventilation efficiency (VE/VCO2 slope) during exercise, likely via different chemoreceptor modulation [69, 70] (Fig. 4). Although the attenuation of hyperventilation could prompt a symptomatic benefit in COPD patients with HFrEF, increased ventilation is a pivotal compensatory mechanism of hypoxia, whose impediment by carvedilol could be detrimental in specific settings, such as COPD patients requiring oxygen therapy [70].

#### β-blockers in COPD without HF

Evidence on the safety and differing prognostic impact of *β*1-selective and non-selective *β*-blockers in COPD comes from large retrospective studies [71–77] and meta-analyses of prospective observational or randomized controlled trials (RCTs) [78–80]. The first important message is that “cardioselective” (*β*1-selective) *β*-blockers have no deleterious effect on airway function [78, 80] and do not change the responsiveness of the FEV_1_ to *β*-agonist administration. Observational studies suggest that *β*1-selective blockers may reduce the risk of exacerbations and death in patients with COPD and no HF [73, 75, 76, 79, 80] but this was not confirmed in a recent RCT [81]. The BLOCK COPD trial was interrupted prematurely because hospitalization for exacerbation was more common among the patients treated with metoprolol. However, at the moment, in COPD patients,*β*1-selective blockers should not be discontinued whenever prescribed for cardiovascular purposes different from HF (angina, atrial fibrillation).

#### β-blockers in COPD with HFrEF

Noteworthy, when HFrEF is the indication for *β*-blockers, evidence indicates a clear benefit on both cardiovascular (CV) and non-CV outcomes (reduction of AECOPD) [71, 72, 74, 77]. In particular, bisoprolol reduces mortality and the incidence of HF and/or COPD exacerbations in a dose-dependent manner compared to carvedilol or metoprolol [72, 74]. A recently published retrospective study based on the Swedish HF Registry confirmed how beta-blocker use in patients with HFrEF and COPD was associated with a lower risk of CV death/total HFH, without evidence of safety concerns for COPD exacerbations [82]. COPD differently impacts EF phenotypes of HF: patients with COPD and HFrEF have a greater risk of HF-specific hospitalization and mortality, while outcomes in patients with comorbid COPD and HFpEF are largely driven by COPD (AECOPD) [9]. Clinicians treating patients with both HF and COPD must carefully weigh the benefits of *β*-blockers for HF (and the actual risk of *β*-blockers withdrawal [83]) against their potential harms to COPD.

**Fig. 5:**
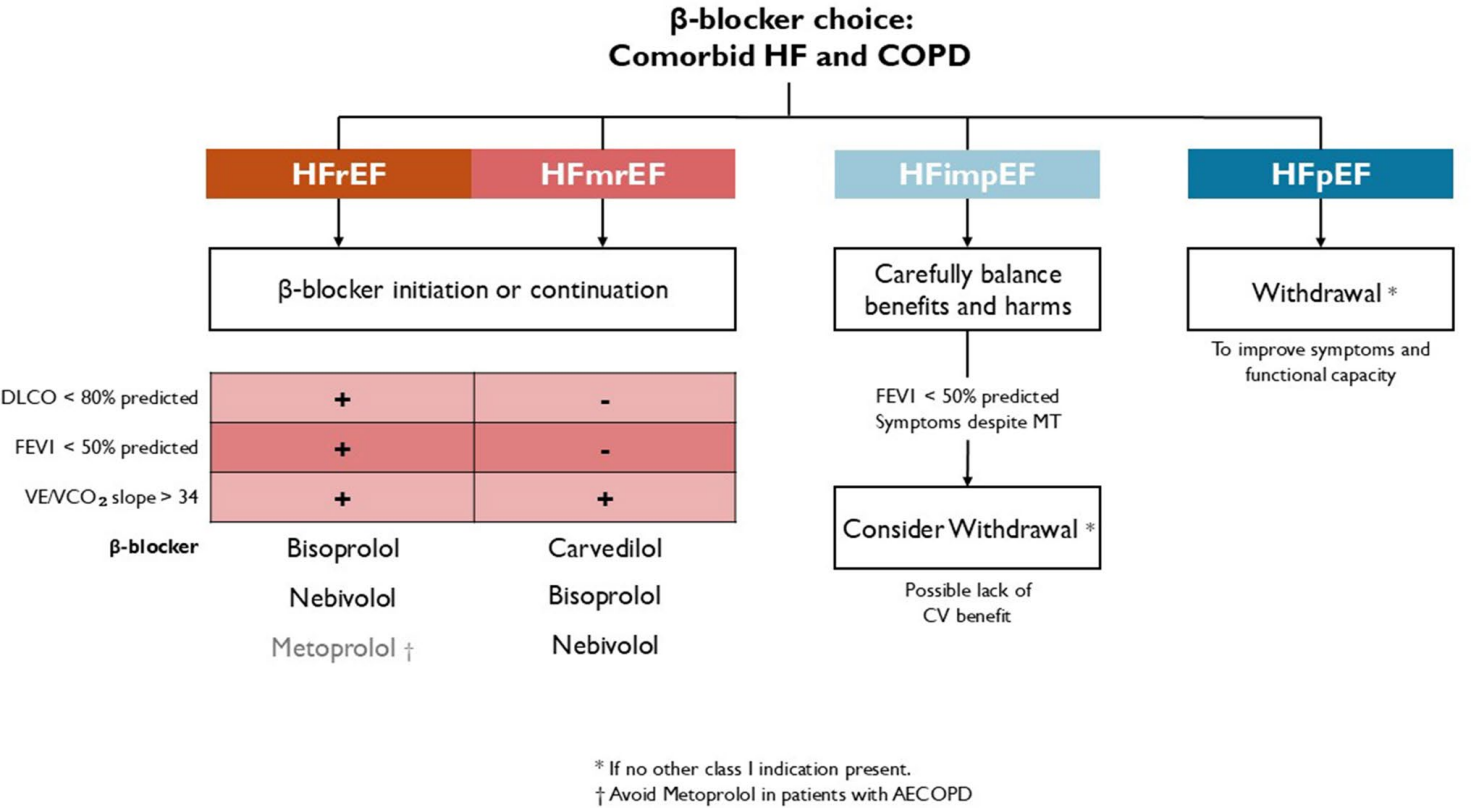
Pragmatic algorithm for the *β*-blocker choice according to HF phenotype, COPD severity, and functional testing (CPET, PFTs). In patients with comorbid HF and COPD, LVEF phenotype is crucial in the decision making regarding *β*-blocker treatment. In patients with HFpEF and COPD, *β*-blockers should be avoided and, when already present, discontinued to improve functional capacity and avoid possible side-effects (unless recommended for non-HF indication). In patients with HF and improved ejection fraction (HFimpEF), evidence of persistent benefit of *β*-blockade once EF normalizes (LVEF > 50%) is limited—the decision to maintain *β*-blockers should be individualized, weighing the severity of airflow obstruction and persistence of respiratory symptoms. In HFrEF patients (LVEF < 40%) with COPD, *β*-blockers remain strongly recommended (Class I, level of evidence A). The choice of the agent should consider the the diffusion capacity (DLCO reduced or need for long-term oxygen therapy), the presence of hyperventilation (VE/VCO_2_ slope increased), the severity of airflow obstruction (FEV1creduced) and the history of COPD exacerbations (AECOPD); FEV1, forced expiratory volume in 1 s; DLCO, diffusing capacity of the lungs for carbon monoxide; VE/VCO2, ventilatory efficiency slope

Overall, *β*1-selective blockers should be preferred in patients with COPD and HFrEF. In the presence of hypoxia (e.g., COPD in oxygen therapy) or reduced diffusion capacity, nebivolol and bisoprolol are the *β*-blockers of choice. In case of an exaggerated ventilatory response during exercise, carvedilol could be considered but should be started at a low dose, and PFT should be repeated after titration [65]. Metoprolol is a second choice in patients with COPD and frequent exacerbations [81]. A pragmatic algorithm for agent selection based on HF phenotype, COPD severity, and functional testing is provided (Fig. 5).

#### β-blockers in COPD with HFpEF/HFimpEF

If, for HFrEF, the conflicting results arising from the paucity of evidence on the detrimental effects of *β*-blockers in COPD must be weighed against one certainty (*β*-blockers markedly improve symptoms and survival), the same cannot be said for HFpEF or HF with improved EF (HFimpEF) [83, 84]. *β*-blocker use in HFpEF is not specifically recommended, could be detrimental [46], and should be avoided, even more so in the presence of COPD. Given the paucity in treatments for HFpEF, better differentiation between cardiac and respiratory symptoms may provide a better management of comorbidities as predisposing and precipitating factors [12]. This paradigm shift could lead to an improvement in quality (if not quantity) of life in this complex and highly comorbid group [85].

### Bronchodilators

Long-acting bronchodilator inhalers are the foundational therapy in COPD [86], relieving symptoms and reducing major outcomes. Bronchodilation is achieved by locally modulating adrenergic tone in smooth muscle cells, either through stimulation of *β*2-adrenergic receptors by long-acting *β*2-agonists (LABAs) or inhibition of M3 cholinergic receptors by long-acting muscarinic antagonists (LAMAs), or both. Overall, long-acting bronchodilators increase the adrenergic nervous system (ANS) tone (directly or indirectly by vagal inhibition), and this pharmacodynamic profile could be particularly unfavorable in the context of HF. Increased sympathetic stimulation on the heart and blood vessels results in vasoconstriction, increased heart rate [87], and possible ischemia, precipitating HF. Compared to normal subjects, failing myocytes present a shift toward *β*2-adrenergic receptors due to *β*1 downregulation [88]. This makes HF muscle even more vulnerable to *β*2 agonists in terms of chronotropic and inotropic responsiveness and raises questions concerning the cardiovascular safety of long-acting bronchodilators. On the other hand, bronchodilators seem to have a promising effect in the cardiorespiratory interaction, breaking the vicious circle of dynamic hyperinflation and cardiac underfilling [89].

#### Safety of bronchodilators in patients without known HF history

Evidence on the net prognostic effect of these therapies in HF patients is scant. Clinical trials on currently used bronchodilators have not demonstrated a significant impact on cardiovascular outcomes [90, 91]. However, while specifically considering patients with established cardiovascular disease, these studies excluded those with symptomatic HF, thereby limiting the relevance of their findings in the COPD–HF overlap scenario.

Several observational studies [92–98] and the most recent meta-analysis [99] have reported conflicting results on the cardiovascular risks associated with the use of long-acting bronchodilators in COPD. In particular, the retrospective analysis of these registries focuses on the impact of LAMA/LABA initiation and exposure. Some authors describe an independent association between LABA (more than LAMA) initiation and the risk of HFH [92–94, 97], with the highest risk within 30 days after incident treatment and a neutral (or even protective) effect for those who were already receiving the medication [96]. A trend towards a CV beneficial effect of long-term exposure to LAMA/LABA has been confirmed by a recent retrospective study [98].

#### Safety of bronchodilators in patients with COPD and HF

Different retrospective studies specifically concentrate on comorbid COPD and HF population [100–102]. The treatment with LABA seems associated with HF exacerbations among COPD patients with known left ventricular dysfunction or a history of HF, in a dose–response relationship [100, 101]. However, the strength of this association is reduced after adjustment for possible confounders and disappears when correcting for smoking status. A rigorous analysis of well-characterized HF patients [102], unlike previous reports, clearly shows no relationship between LABA and long-term outcomes when adjusted for population differences, including BNP.

These conflicting results arise from the inherent biases of retrospective observational cohort studies, where treatment purchase is equated with actual treatment, and diagnoses rely on insurance codes and labels. Misdiagnosis between HFH and AECOPD is indeed another limitation when interpreting the results of these studies [103]. Any retrospective association between a treatment and subsequent CV events is undermined by the indication for LABA/LAMA use: increasing dyspnea and resulting beta-agonist prescription may simply reflect worsening HF [84]. Besides, all these retrospective studies lack COPD (and even more HF) disease severity stratification. Is medication intensity or COPD severity related to HF events? The poor outcomes attributed to beta-agonists may reflect the disease for which they are prescribed, and separating the two is difficult [104]. A randomized controlled trial on LABA/LAMA in COPD patients with concomitant HF, focused on HF-specific safety outcomes, is needed to definitely dissolve this old myth.

Although concerns about the use of inhaled bronchodilators persist, evidence-based COPD treatment should be offered irrespective of HF status [47]. At present, the combination of a LAMA and an inhaled corticosteroid (ICS) may be preferred for HF patients newly diagnosed with COPD, or vice versa [99]. It can be speculated that concurrent treatment with *β*1-selective blockers, by promoting the re-exposure of *β*1-adrenergic receptors and reducing *β*2 dependence [105], could mitigate LABA-related side effects; however, supporting evidence for this hypothesis is lacking.

## Conclusions

The coexistence of COPD and HF presents significant clinical challenges, exacerbating morbidity and complicating diagnosis and treatment. This review highlights the intricate bidirectional pathophysiological interactions between these conditions, emphasizing the impact of pulmonary congestion on airway function and the detrimental effects of COPD-related hyperinflation on cardiac performance. Despite a plausible pathophysiologic rationale, the impact of dynamic hyperinflation on exercise physiology in patients with HF has not been extensively studied and should merit further characterization.

The differential diagnosis of HF and COPD remains complex due to overlapping symptoms and diagnostic limitations, with natriuretic peptides and pulmonary function tests providing only partial clarity. Further studies validating a disease-specific cut-off for NPs testing in comorbid COPD and HF, and assessing the prognostic implications of CPET-derived VE intercept, are the main research perspectives in the diagnostic field.

The therapeutic management of these comorbidities requires a nuanced approach, particularly regarding *β*-blocker use. While *β*1-selective blockers like nebivolol and bisoprolol improve cardiovascular outcomes and reduce COPD exacerbations, non-selective agents like carvedilol may impair pulmonary function, and its use should be restricted to selected patients. The role of bronchodilators in HF remains controversial, with conflicting evidence regarding their cardiovascular safety: in this field, a RCT comparing LABA and LAMA, focused on safety in HF-related outcomes represent a significant gap in evidence.

## Supplementary Information

Below is the link to the electronic supplementary material.

ESM 1 (PPTX 431 KB)

## Data Availability

No datasets were generated or analyzed during the current study.
