# Supplementary material for: Heart failure and chronic obstructive pulmonary disease. A combination not to be underestimated
Source: Heart Fail Rev. 2025 Oct 7;30(6):1525–38. doi: 10.1007/s10741-025-10566-3 (PMC12618358; doi:10.1007/s10741-025-10566-3)
Supplement: Supplementary file 1 — (PPTX 431 KB) [file 10741_2025_10566_MOESM1_ESM.pptx]

## Slide 1
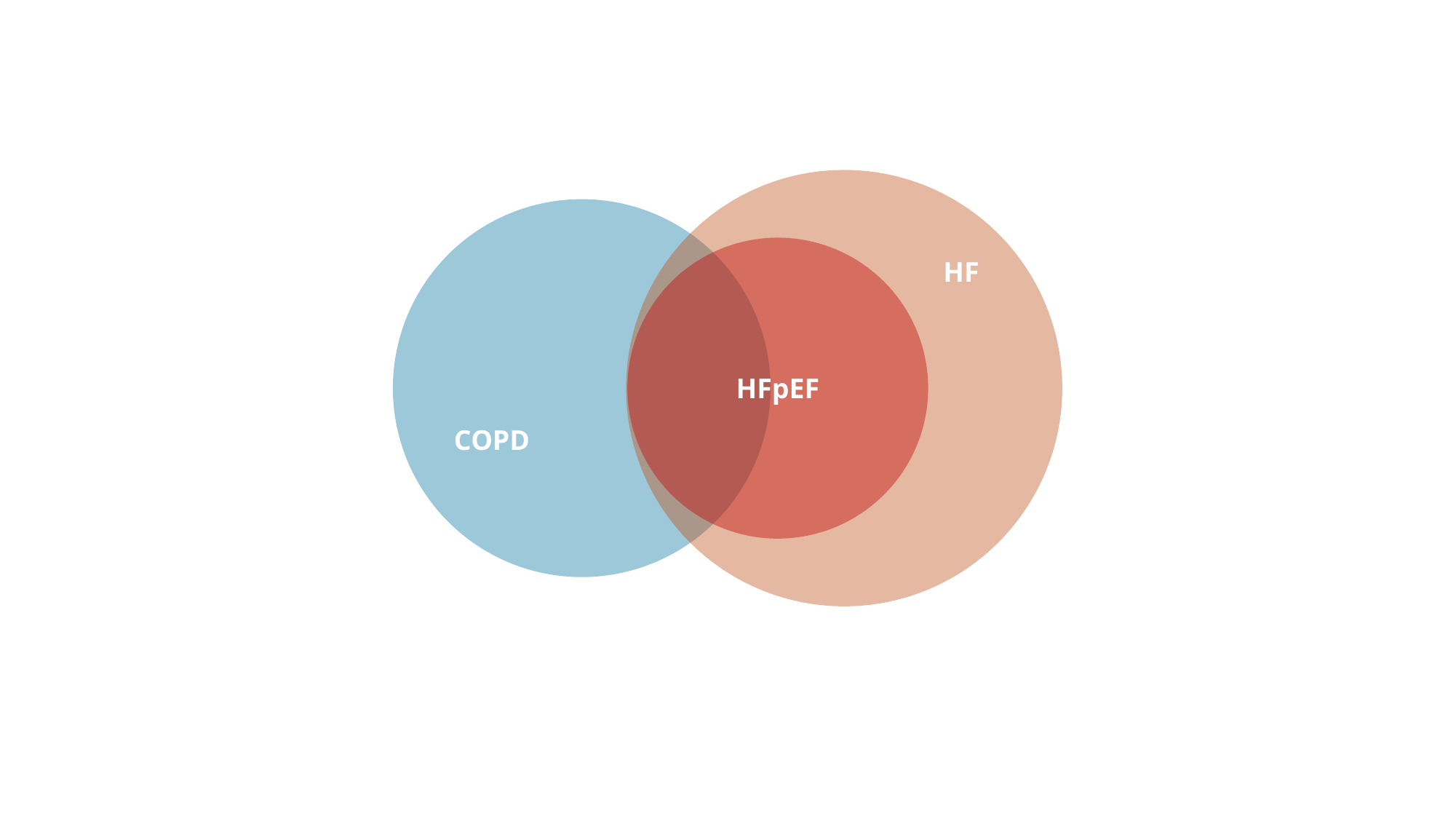

HF
HFpEF
COPD

## Slide 2
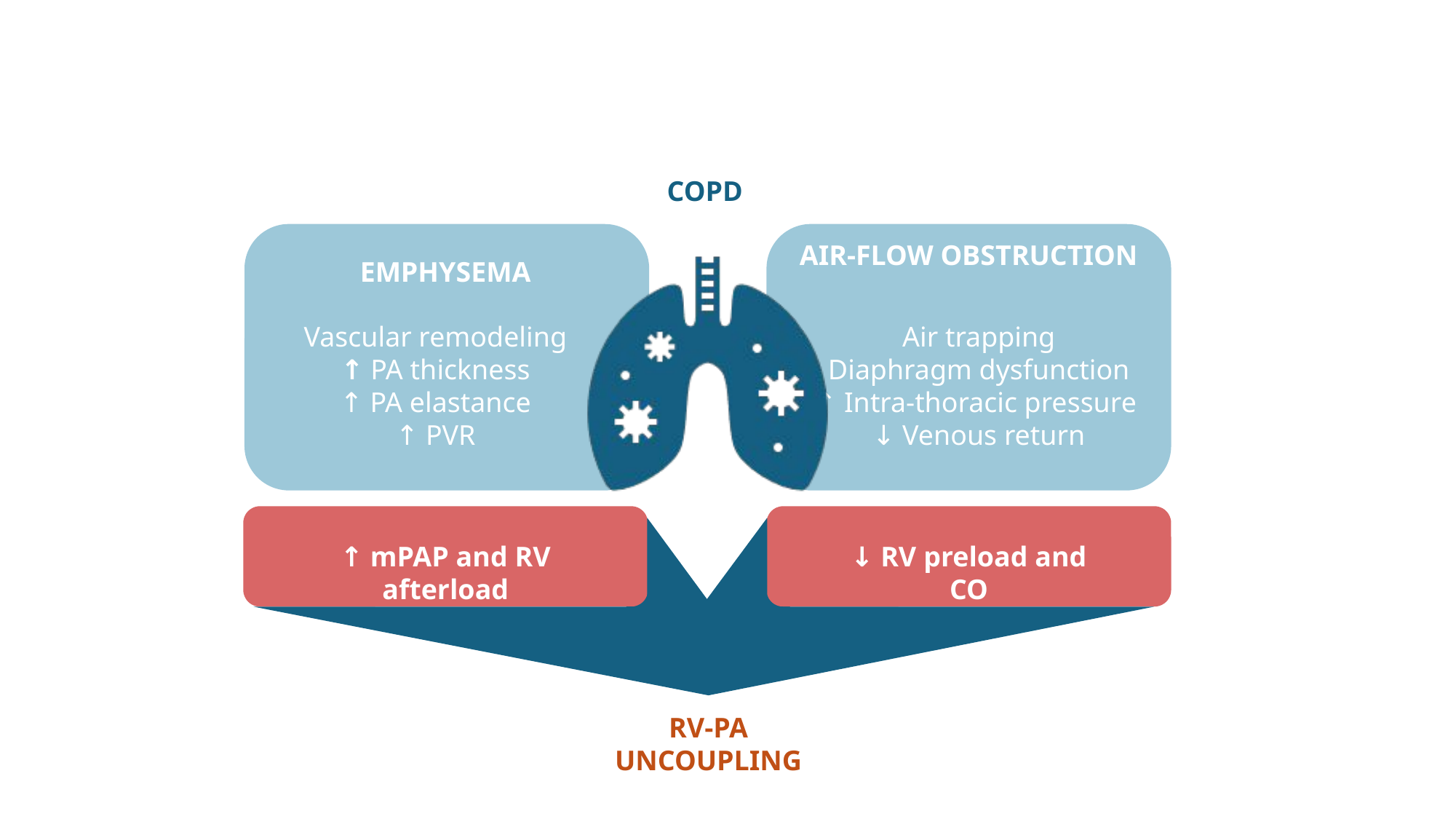

COPD
AIR-FLOW OBSTRUCTION
EMPHYSEMA
Air trapping
Diaphragm dysfunction
↑ Intra-thoracic pressure
↓ Venous return
Vascular remodeling
↑ PA thickness
↑ PA elastance
↑ PVR
↑ mPAP and RV afterload
↓ RV preload and CO
RV-PA UNCOUPLING

## Slide 3
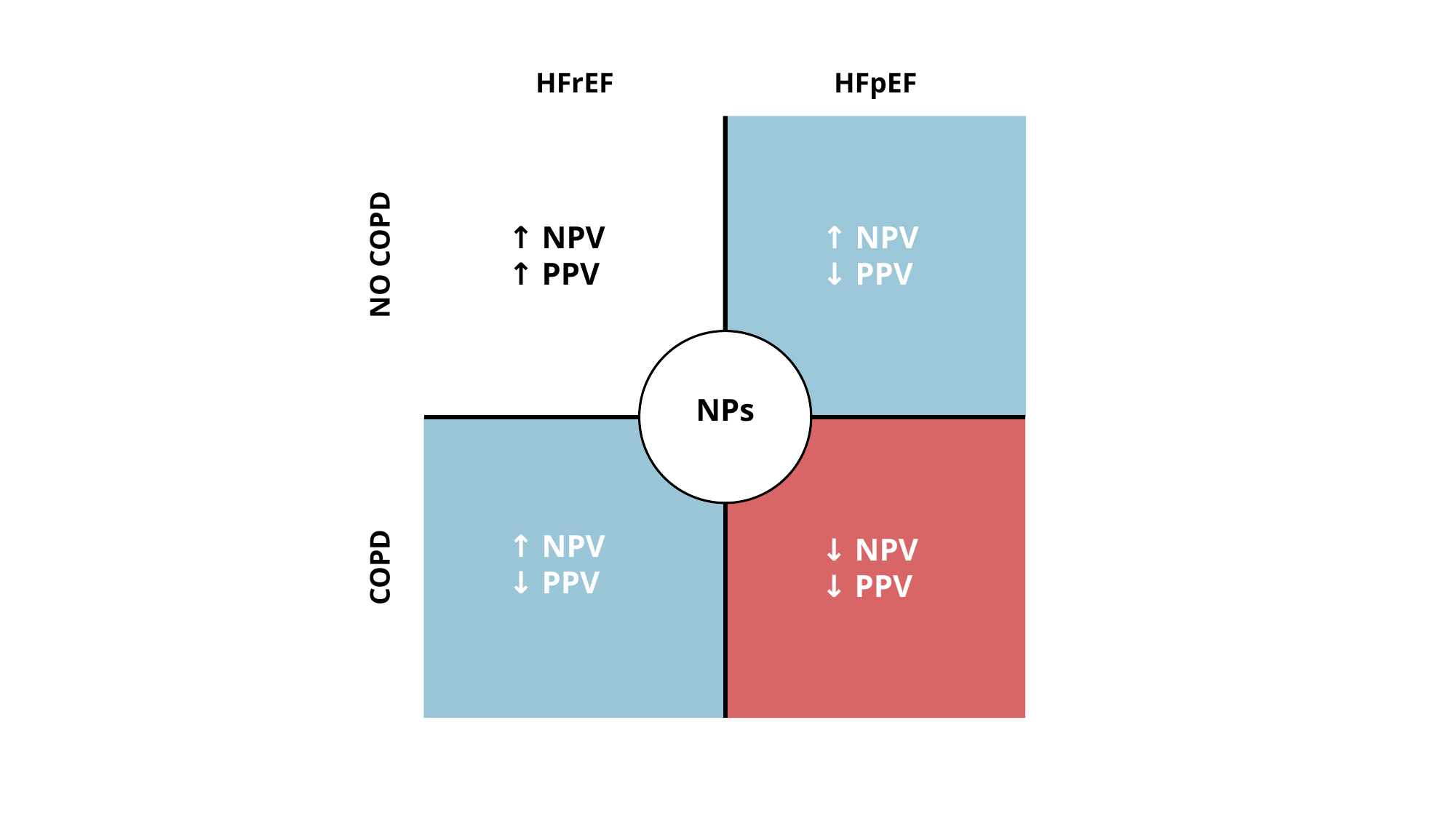

HFrEF
HFpEF
↑ NPV
↑ PPV
↑ NPV
↓ PPV
NO COPD
NPs
↑ NPV
↓ PPV
↓ NPV
↓ PPV
COPD

## Slide 4
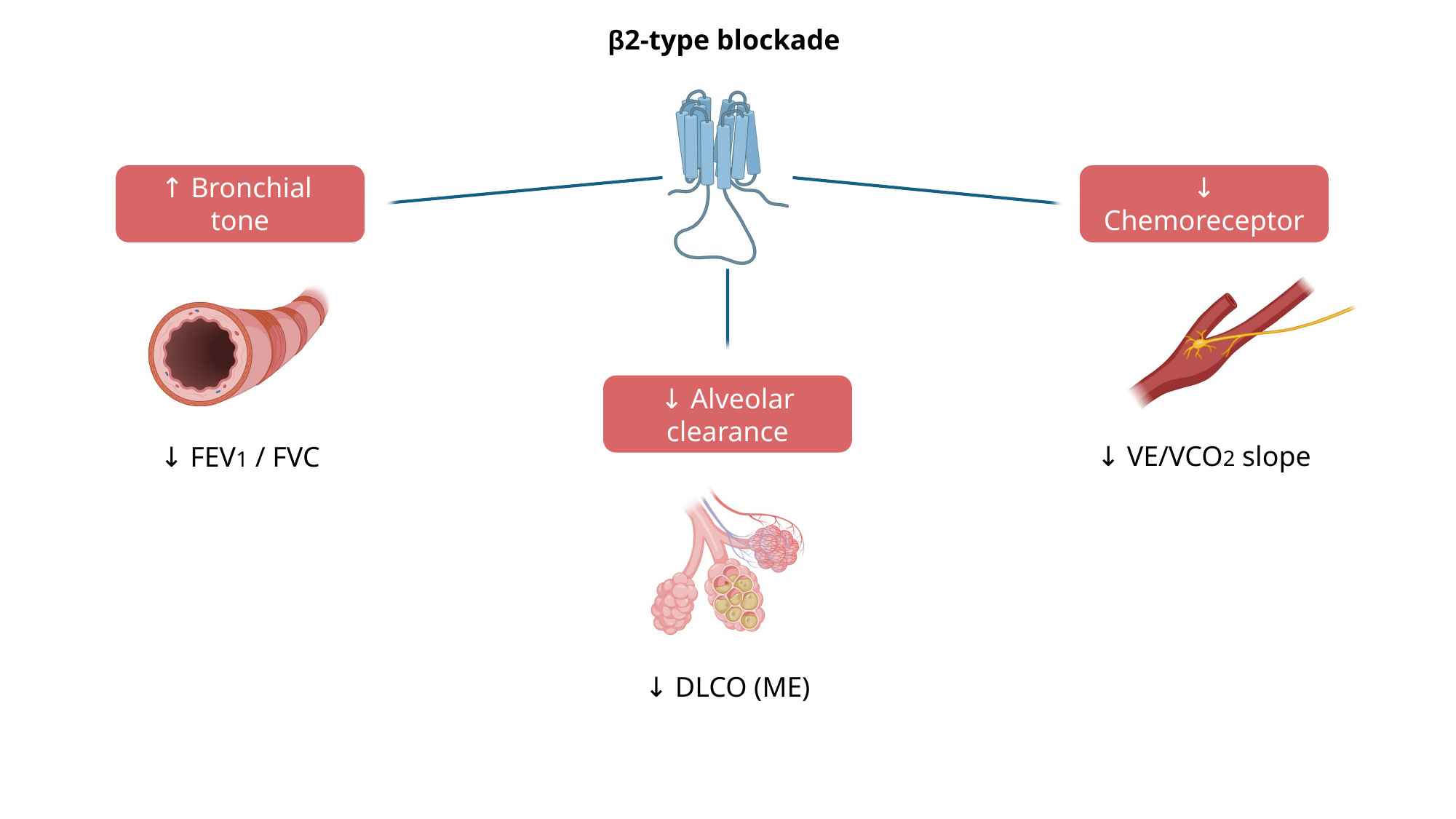

β2-type blockade
↑ Bronchial
tone
↓ Chemoreceptor sensitivity
↓ Alveolar clearance
↓ VE/VCO2 slope
↓ FEV1 / FVC
↓ DLCO (ME)

## Slide 5
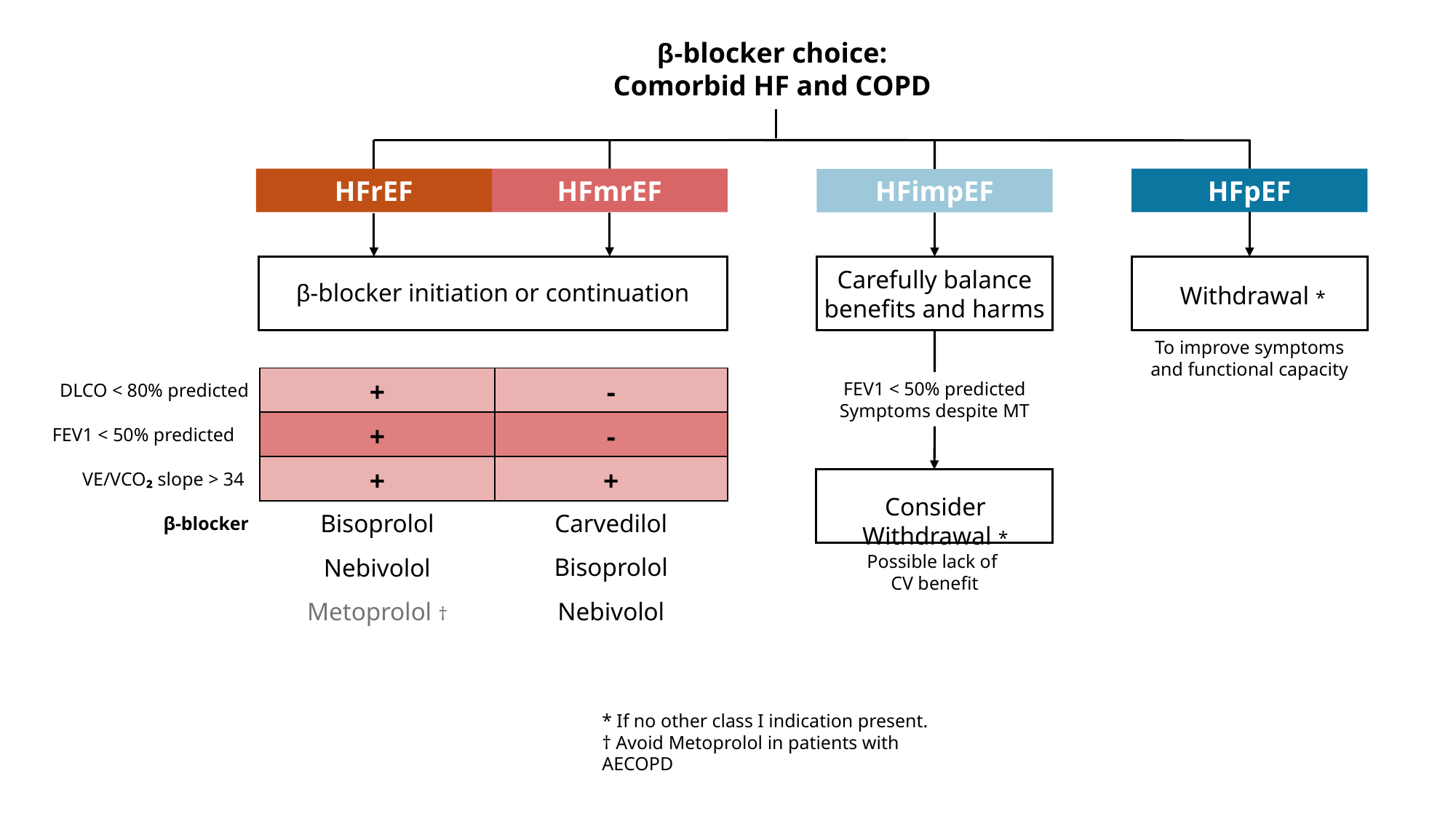

β-blocker choice:
Comorbid HF and COPD
HFrEF
HFmrEF
HFpEF
HFimpEF
Carefully balance benefits and harms
β-blocker initiation or continuation
Withdrawal *
To improve symptoms and functional capacity
| DLCO < 80% predicted | + | - |
| --- | --- | --- |
| FEV1 < 50% predicted | + | - |
| VE/VCO₂ slope > 34 | + | + |
| β-blocker | Bisoprolol | Carvedilol |
| | Nebivolol | Bisoprolol |
| | Metoprolol † | Nebivolol |
FEV1 < 50% predicted
Symptoms despite MT
Consider Withdrawal *
Possible lack of
CV benefit
* If no other class I indication present.
† Avoid Metoprolol in patients with AECOPD
